# Prognostic significance of pre- and post-treatment hematological biomarkers in patients with head and neck cancer treated with chemoradiotherapy

**DOI:** 10.1038/s41598-023-30584-1

**Published:** 2023-03-08

**Authors:** Yoh-ichiro Iwasa, Moeka Shimizu, Kazuki Matsuura, Kentaro Hori, Ken Hiramatsu, Kenjiro Sugiyama, Yoh Yokota, Tomohiro Kitano, Ryosuke Kitoh, Yutaka Takumi

**Affiliations:** 1 Department of Otorhinolaryngology-Head and Neck Surgery, Shinshu University School of Medicine, 3-1-1, Asahi, Matsumoto City, 390-8621 Japan; 2 Shinshu University School of Medicine, 3-1-1, Asahi, Matsumoto City, 390-8621 Japan

**Keywords:** Head and neck cancer, Tumour biomarkers

## Abstract

This study aimed to investigate the prognostic value of hematological biomarkers measured before and after treatment in patients with head and neck cancer (HNC). This study reviewed 124 patients with HNC who received chemoradiotherapy. Hematological biomarkers assessed before and after treatment were investigated. The pretreatment C-reactive protein/albumin ratio (pre-CAR) and post-treatment prognostic nutritional index (post-PNI) showed the highest area under the curve with cutoff values of 0.0945 and 34.9, respectively. Patients in the high pre-CAR group showed significantly worse prognosis than those in the low pre-CAR group with respect to the progression-free survival (PFS) (3-year PFS: 44.8% vs. 76.8%, *p* < 0.001) and overall survival (OS) (3-year OS: 65.8% vs. 94.0%, *p* < 0.001). Patients in the low post-PNI group showed significantly worse prognosis than those in the high post-PNI group with respect to the PFS (3-year PFS: 58.6% vs. 77.4%, *p* = 0.013) and OS (3-year OS: 75.2% vs. 96.9%, *p* = 0.019). Multivariate analysis revealed that advanced N stage (*p* = 0.008), high pre-CAR (*p* = 0.024), and low post-PNI (*p* = 0.034) were significantly associated with poorer OS. We suggest that the evaluation of hematological markers before and after treatment is useful for predicting disease progression and survival.

## Introduction

Head and neck cancer (HNC), which is the seventh most common malignancy in the world^1^, involves several subsites, such as the oral cavity, oropharynx, hypopharynx, larynx, nasopharynx, salivary gland, and nasal/paranasal cavity. Estimates indicate that 562,328 people were newly diagnosed with HNC in 2020 worldwide^2^. The major histological type of HNC is squamous cell carcinoma (SCC), which accounts for over 90% of cases. Surgery-based treatment is mainly indicated in patients with salivary gland cancer, oral cancer, and locally advanced tumors, while radiotherapy-based treatment is mainly indicated for nasopharyngeal cancer and HPV-positive oropharyngeal cancer, and is also an important treatment strategy for preservation of the larynx, especially in patients with hypopharyngeal and laryngeal cancers.

Inflammatory biomarkers, such as the ﻿neutrophil/lymphocyte ratio (NLR), platelet/lymphocyte ratio (PLR), and lymphocyte/monocyte ratio (LMR), have been reported as ﻿prognostic markers for various cancers, including HNC^3–6^. Recently, the C-reactive protein (CRP)/albumin ratio (CAR) and other nutritional markers, such as the prognostic nutritional index (PNI) and prognostic immune and nutritional index (PINI), reportedly possess prognostic value for many types of malignancies^7–12^.

Although these hematological biomarkers are readily measured in clinical settings and are reported to be reliable prognostic markers of various malignancies, the markers that reflect the prognosis in patients with HNC most precisely remain to be elucidated. Moreover, previous studies mainly evaluated these biomarkers before treatment, and little is known about the significance of the post-treatment evaluation of these markers in HNC. The current study aimed to investigate the prognostic value of NLR, LMR, PLR, CAR, PNI, and PINI before and after treatment in patients with HNC. In this retrospective study, we report the results of 124 patients with HNC who underwent chemoradiotherapy (CRT) at our institution.

## Results

### Patient characteristics

﻿The clinicopathological characteristics of the 124 patients are presented in Table 1. Their median age was 64.5 (range, 34–76) years. The median follow-up period was 45.3 (range: 11–93) months. Seventeen patients underwent a short follow-up of less than 24 months. A total of 103 patients were men (83.1%) and 21 patients were women (16.9%). At the time of the first visit, 47 (37.9%) participants were current tobacco users. p16 immunohistochemistry was positive in 38 patients (30.6%) and negative or unknown in 86 patients (69.4%). The primary tumor sites were the oropharynx in 47 patients (37.9%), hypopharynx in 31 patients (25.0%), larynx in 18 patients (14.5%), nasopharynx in 20 patients (16.1%), and nasal cavity/maxillary sinus in 8 patients (6.5%). Among the 20 patients with nasopharyngeal carcinoma, 12 tested positive for the Epstein-Barr virus (EBV), and the EBV infection status was negative or unknown in 8 patients. Stage I disease occurred in 27 patients (21.8%), stage II in 31 patients (25.0%), stage III in 26 patients (21.0%), and stage IV in 40 patients (32.3%). The majority of patients (n = 116; 93.5%) had SCC, while 8 patients (6.5%) had lymphoepithelial carcinoma.

**Table 1:** Clinical characteristics of the 124 patients included in this study.

Variables		n (%)	pre-CAR (median)	post-PNI (median)
Age (years)	Median (range)	64.5 (34–76)	0.026	36.1
Sex	Male	103 (83.1)	0.026	36.3
	Female	21 (16.9)	0.025	34.7
ECOG PS	0	114 (91.9)	0.024	36.5
	1	10 (8.1)	0.091	33.7
Smoking	Absent	77 (62.1)	0.024	36.5
	Present	47 (37.9)	0.026	34.5
p16 status	Positive	38 (30.6)	0.020	37.3
	Negative/unknown	86 (69.4)	0.028	34.6
Primary site	Oropharynx	47 (37.9)	0.023	36.7
	Hypopharynx	31 (25.0)	0.029	34.5
	Larynx	18 (14.5)	0.022	36.3
	Nasopharynx	20 (16.1)	0.021	34.5
	Nasal cavity/maxillary sinus	8 (6.5)	0.031	36.5
T category	Tx	1 (0.1)	0.088	37.4
	T1	18 (14.5)	0.024	35.5
	T2	65 (52.4)	0.014	37.2
	T3	24 (19.4)	0.043	34.1
	T4	16 (12.9)	0.098	33.3
	N0	32 (25.8)	0.018	36.8
N category	N1	44 (35.5)	0.019	36.5
	N2	43 (34.7)	0.036	34.1
	N3	5 (4.0)	0.238	33.3
Stage	I	27 (21.8)	0.019	37.5
	II	31 (25.0)	0.023	37.7
	III	26 (21.0)	0.024	34.1
	IV	40 (32.3)	0.057	34.1
Histology	SCC	116 (93.5)	0.026	36.3
	LEC	8 (6.5)	0.009	34.0

*CAR* C-reactive protein/albumin ratio, *PNI* prognostic nutritional index, *SCC* squamous cell carcinoma, *LEC* lymphoepithelial carcinoma.

### ﻿Cutoff values for pre- and post-treatment markers

**Figure 1:**
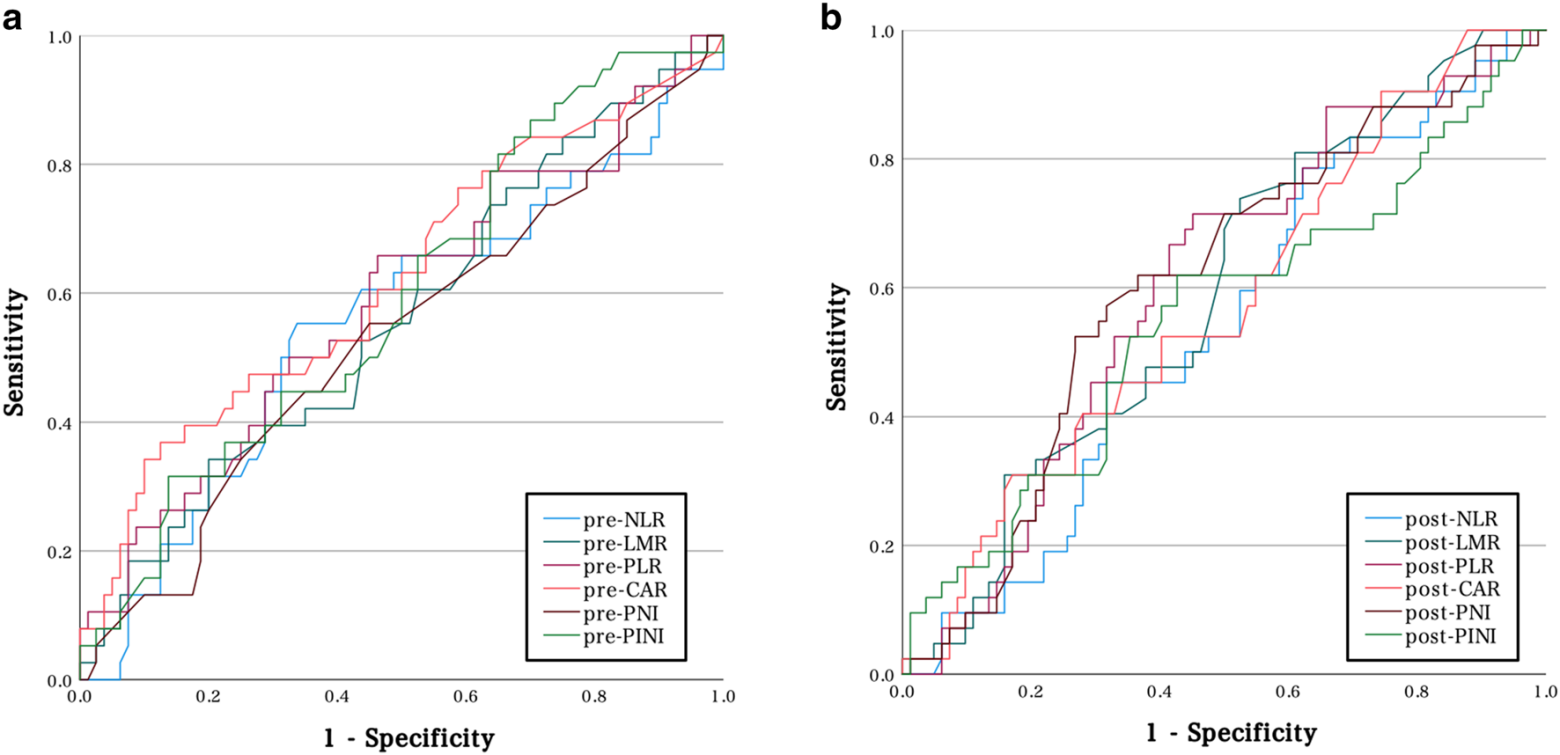
Receiver operating characteristic (ROC) curves for progression-free survival (PFS) according to the pre- and post-treatment markers.

﻿Receiver operating characteristic (ROC) curve analysis was performed, and the area under the curve (AUC) was calculated to assess the discriminatory ability of pre- and post-treatment hematological biomarkers (Fig. 1). The AUCs of the pretreatment biomarkers, viz. pre-NLR, pre-LMR, pre-PLR, pre-CAR, pre-PNI, and pre-PINI, were 0.556, 0.560, 0.589, 0.621, 0.534, and 0.592, respectively. The optimal cutoff value of pre-CAR, which showed the highest AUC among the pretreatment biomarkers, was determined to be 0.0945 (sensitivity, 36.8%; specificity, 87.5%; negative predictive value, 73.0%; positive predictive value, 60.2%) with reference to Youden’s index. The AUCs of the post-treatment biomarkers, viz. post-NLR, post-LMR, post-PLR, post-CAR, post-PNI and post-PINI were 0.538, 0.589, 0.603, 0.573, 0.608, and 0.592, respectively. Post-PNI showed the highest AUC, with a cut-off value of 34.9 (sensitivity, 61.9%; specificity, 62.2%; negative predictive value, 76.1%; positive predictive value, 45.6%).

### Survival analysis according to pre-CAR and post-PNI

**Figure 2:**
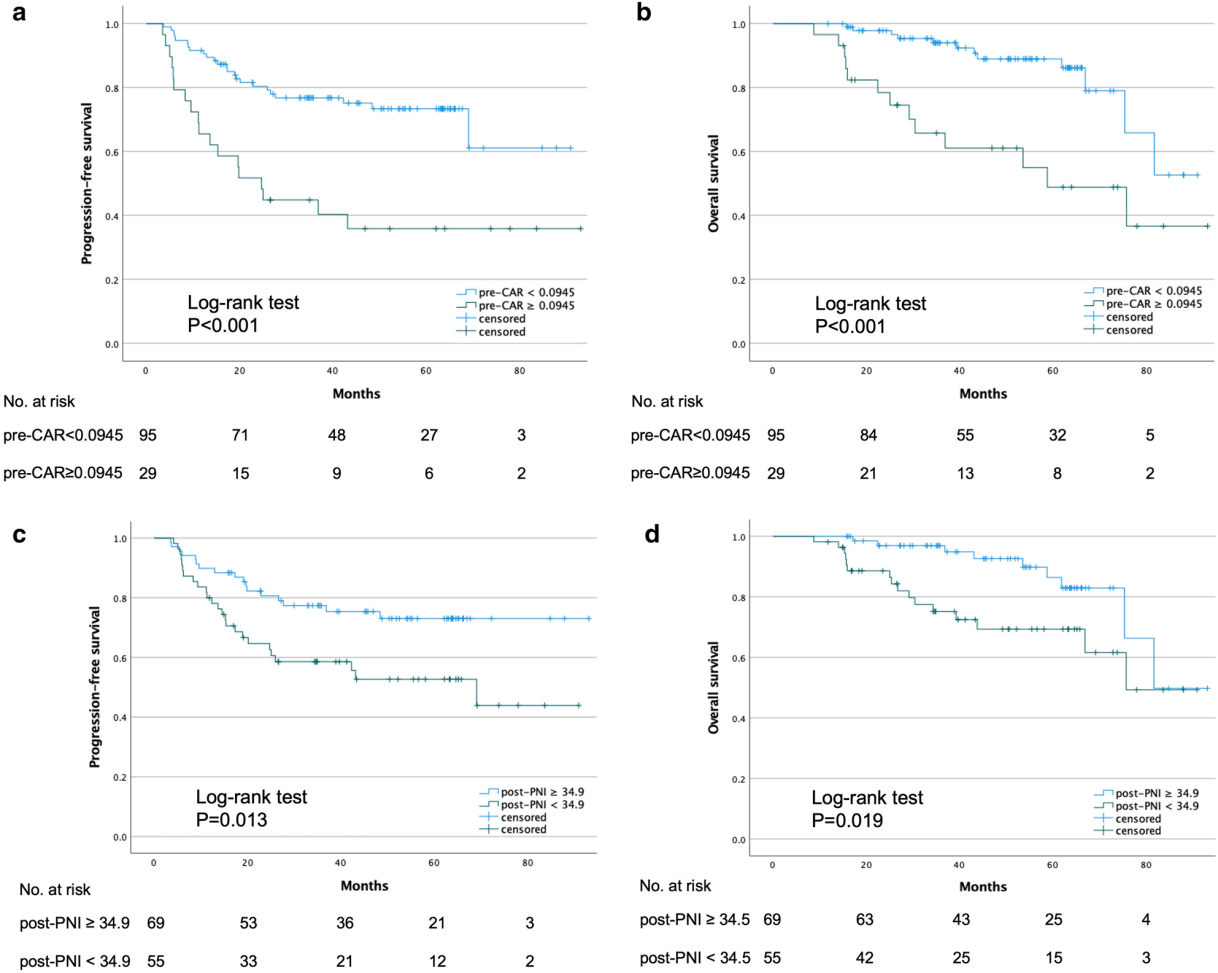
Kaplan–Meier curves for progression-free survival (PFS) and overall survival (OS) according to the pre-CAR (**a**,**b**) and post-PNI (**c**,**d**).

Patients were divided into two groups, according to the pre-CAR and post-PNI cutoff values. The Kaplan–Meier curves for progression-free survival (PFS) and overall survival (OS) plotted according to the pre-CAR and post-PNI are presented in Fig. 2. Patients in the high pre-CAR (pre-CAR ≥ 0.0945) group exhibited a significantly worse prognosis than those in the low pre-CAR (pre-CAR < 0.0945) group with respect to PFS (3-year PFS: 44.8% vs. 76.8%, *p* < 0.001) and OS (3-year OS: 65.8% vs. 94.0%, *p* < 0.001). Patients in the low post-PNI (post-PNI < 34.9) group had a significantly worse prognosis than those in the high post-PNI (post-PNI ≥ 34.9) group with respect to the PFS (3-year PFS: 58.6% vs. 77.4%, *p* = 0.013) and OS (3-year OS: 75.2% vs. 96.9%, *p* = 0.019).

### Prognostic factors for PFS and OS

﻿The effects of the background factors on PFS and OS are depicted in Table 2. Univariate analysis revealed that p16 status (*p* = 0.016), advanced T stage (*p* = 0.004), advanced N stage (*p* < 0.001), pre-CAR (*p* < 0.001), and post-PNI (*p* = 0.015) were associated with poorer PFS. Multivariate analysis revealed that only the advanced N stage (*p* < 0.001) was significantly associated with poorer PFS. Univariate analysis of OS revealed that advanced T stage (*p* = 0.033), advanced N stage (*p* < 0.001), pre-CAR (*p* < 0.001), and post-PNI (*p* = 0.024) were associated with poorer OS. Multivariate analysis showed that advanced N stage (*p* = 0.008), pre-CAR (*p* = 0.024), and post-PNI (*p* = 0.034) were significantly associated with poorer OS.

**Table 2:** Univariate and multivariate analyses of overall survival and progression-free survival.

	Progression-free survival				Overall survival			
	Univariate analysis		Multivariate analysis		Univariate analysis		Multivariate analysis	
	Hazard ratio (95% CI)	*p*-value	Hazard ratio (95% CI)	*p*-value	Hazard ratio (95% CI)	*p*-value	Hazard ratio (95% CI)	*p*-value
Age (years)								
< 70	1				1			
≥ 70	0.940 (0.462–1.912)	0.864			1.111 (0.441–2.795)	0.824		
Sex								
Male	1				1		1	
Female	1.613 (0.791–3.290)	0.188			0.343 (0.079–1.479)	0.151	0.229 (0.050–1.044)	0.057
ECOG PS								
0	1				1			
1	2.873 (1.191–6.929)	0.019*			2.664 (0.605–11.725)	0.195		
Smoking								
Absent	1				1			
Present	0.862 (0.459–1.621)	0.645			1.317 (0.597–2.905)	0.495		
p16 status								
Positive	1				1			
Negative/unknown	2.726 (1.210–6.142)	0.016*			2.901 (0.991–8.497)	0.052		
T classification								
T1, T2	1		1		1			
T3, T4	2.430 (1.323–4.463)	0.004*	1.817 (0.964–3.425)	0.065	2.354 (1.072–5.169)	0.033*		
N classification								
N0, N1	1		1		1		1	
N2, N3	4.506 (2.364–8.589)	< 0.001*	3.668 (1.883–7.146)	< 0.001*	4.954 (2.066–11.876)	< 0.001*	3.370 (1.374–8.266)	0.008*
Pre-CAR								
< 0.0945	1		1		1		1	
≥ 0.0945	3.153 (1.705–5.829)	< 0.001*	1.901 (0.990–3.653)	0.054	3.843 (1.736–8.507)	< 0.001*	2.565 (1.131–5.821)	0.024*
Post-PNI								
≥ 34.9	1				1		1	
< 34.9	2.146 (1.156–3.982)	0.015*			2.569 (1.131–5.829)	0.024*	2.515 (1.073–5.895)	0.034*

### Prognostic investigation by the combination of pre-CAR and post-PNI

**Figure 3:**
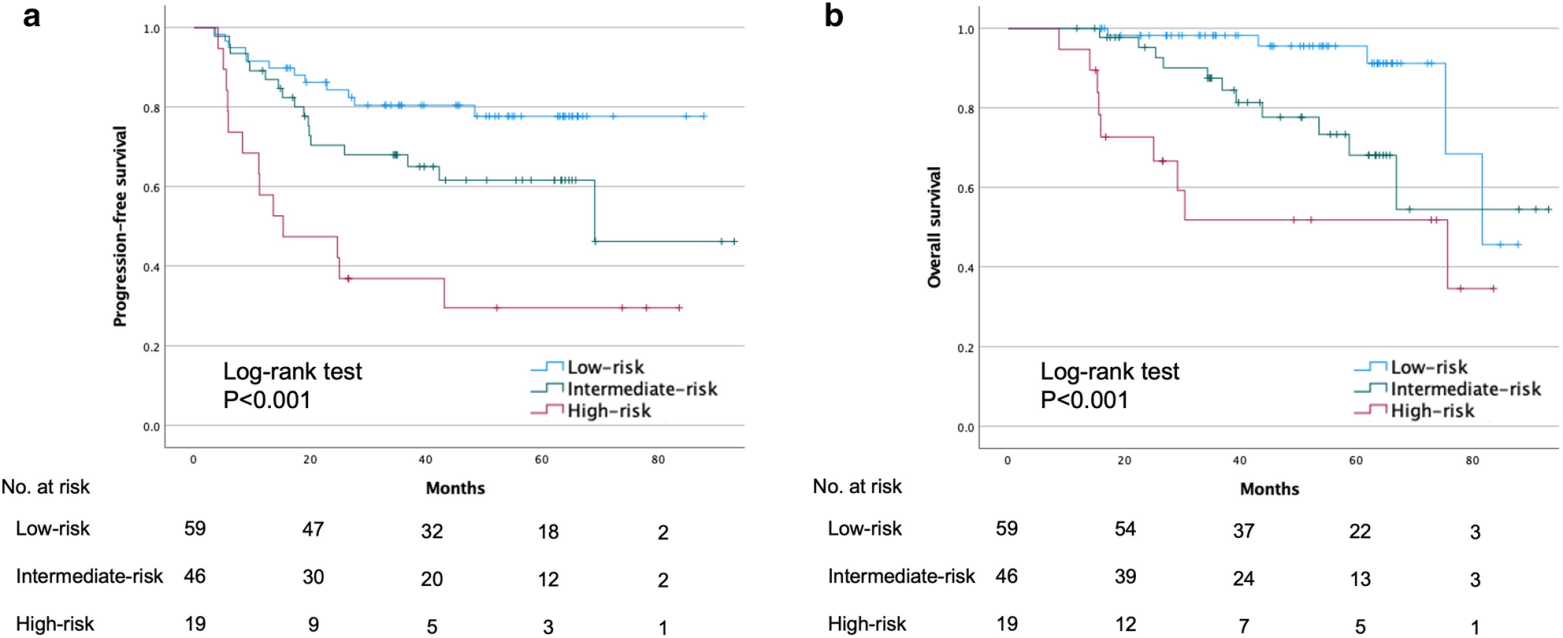
Progression-free survival (PFS) and overall survival (OS) according to the pre-CAR and post-PNI.

As the multivariate analysis indicated that pre-CAR and post-PNI were prognostic factors, we divided the patients into three groups according to their pre-CAR and post-PNI status. Patients with both low pre-CAR and high post-PNI were assigned to the low-risk group, those with either high pre-CAR or low post-PNI were assigned to the intermediate-risk group, and those with both high pre-CAR and low post-PNI were assigned to the high-risk group. The Kaplan–Meier curves for PFS and OS according to the risk groups are presented in Fig. 3. The PFS and OS rates differed significantly among the three groups (*p* < 0.001).

## Discussion

In this study, we evaluated 124 patients with HNC treated with CRT at our institution and aimed to investigate the prognostic value of hematological biomarkers including NLR, LMR, PLR, CAR, PNI, and PINI. Notably, we evaluated these markers not only before treatment but also after treatment. This study revealed that the pre-CAR, a pretreatment biomarker, showed the highest AUC value, and a high pre-CAR was significantly associated with a poor prognosis. The CAR is a hematological marker calculated from the CRP and albumin levels. CRP is an acute reactive protein mainly produced in hepatocytes, which is regulated by proinflammatory cytokines such as interleukin 6, which contributes to the tumor microenvironment, and supports tumor angiogenesis, proliferation, growth, and metastasis^13,14^. Albumin is an indicator of the host’s nutritional status. Therefore, the CAR is thought to reflect not only inflammation, but also the nutritional status of patients with cancer^15^. Akin to our study, the AUC of the CAR is reportedly higher than that of other markers in hepatocellular, colorectal, gastric, and esophageal cancer^13,15–17^. Yamagata et al. investigated the prognostic value of the NLR, PLR, LMR, systemic inflammation response index (SIRI), systemic immune-inflammation index (SII), and CAR for patients with oral cancer, and the AUC was the highest for the CAR^7^. Therefore, the prognostic ability of the CAR could be superior to that of other hematological biomarkers for HNC and other types of cancer.

Although most previous studies have reported the prognostic significance of hematological biomarkers evaluated before treatment, this study showed that the post-treatment evaluation of these markers could also have a prognostic benefit. In this study, a low post-PNI was significantly associated with worse OS in both univariate and multivariate analyses. The PNI is a hematological marker based on the albumin and lymphocyte count, reflecting the nutritional and inflammatory status of the host. A previous study that investigated 107 cases of nasopharyngeal cancer identified both pre- and post-treatment PNI as independent prognostic markers, and the authors suggested that dynamic changes in PNI were more important than the pre-clinical values alone^18^. Since malnutrition is associated with cancer progression^19^, it is plausible that both pre- and post-treatment nutritional conditions are relevant to the prognosis of cancer treatment. Interestingly, the pre-CAR, which was identified as the most reliable pretreatment marker in this study, is a nutritional as well as inflammatory marker, highlighting the importance of nutritional intervention during treatment from the perspective of patient prognosis. The relationship between the dynamics of nutritional status during treatment and patient prognosis warrants further investigation.

As shown in Fig. 3, the combination of the pre-CAR and post-PNI successfully stratified the patients with respect to disease progression and survival. Although we investigated the changes in these markers before and after treatment, we could not find any clear relationship between these changes and the prognosis (Figure S2). Kano et al. suggested that the combination of inflammatory parameters (NLR, LMR, and PLR) was a more sensitive marker^20^. In addition, the combination of NLR and fibrinogen (F-NLR) has been reported to be a prognostic marker in patients with esophageal SCC and hypopharyngeal cancer^21,22^. A study on oral cancer observed significant differences in the OS when patients were stratified according to CAR, stage, and age^7^. Although hematological markers alone are useful clinical predictive markers, a combination of these markers or combinations of hematological markers with other clinical factors could be more sensitive. Recently, hematological biomarkers and dynamic changes in their levels have been reported to be predictors of response to immunotherapy in several malignancies, including HNC^23–26^. Further research on the relationship between hematological markers and cancer therapy is warranted.

This study had some limitations. First, it was a single-center, retrospective study with a small sample size. Thus, there could be potential for selection bias, and the findings of this study should be validated in larger cohorts. Second, although we evaluated post-treatment markers 1 week after treatment, treatment-related inflammation might have affected the results of these markers. In a study on nasopharyngeal cancer, post-treatment PNI was evaluated 1 month after treatment to exclude treatment-related effects^18^. The appropriate timing for post-treatment marker evaluation should be discussed in future research.

In conclusion, we evaluated the hematological markers of 124 patients with HNC treated with CRT at our institution and found that high pre-CAR and low post-PNI were significantly associated with a poor prognosis. We suggest that the evaluation of hematological markers before and after treatment is useful for predicting disease progression and survival in HNC patients treated with CRT.

## Materials and methods

### Study population and data collection

**Figure 4:**
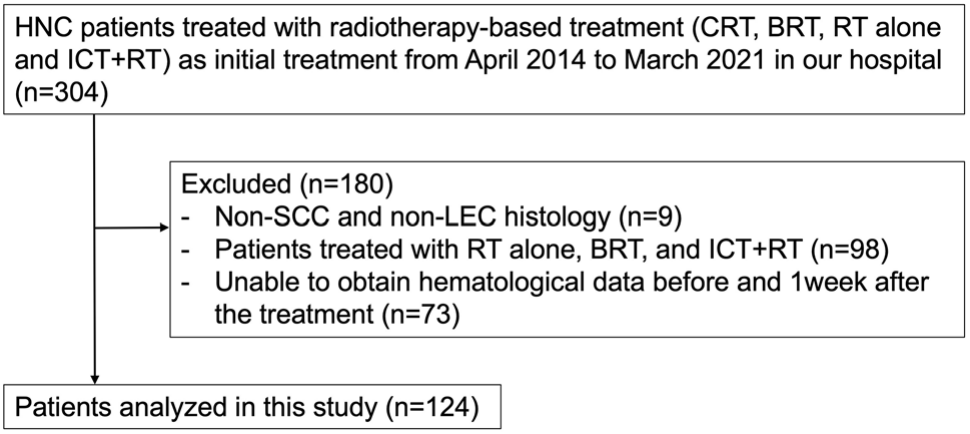
Flow diagram of patient selection. HNC head and neck cancer, CRT chemoradiotherapy, BRT bioradiotherapy; RT radiotherapy, ICT induction chemotherapy, SCC squamous cell carcinoma, LEC lymphoepithelial carcinoma.

This retrospective cohort study enrolled patients with HNC, with the exception of those with oral and salivary gland cancer, who underwent treatment at Shinshu University Hospital between January 2014 and March 2021. Patients who underwent surgical treatment were excluded from this cohort. ﻿One hundred and twenty-four patients who received CRT and whose pre- and post-treatment hematological data were available were reviewed (Fig. 4). ﻿The clinicopathological data of the patients, including age, sex, Eastern Cooperative Oncology Group performance status (ECOG PS), current smoking status, primary tumor site, clinical stage (according to the 8th edition of the TNM classification), histology, p16 status, and hematological data were obtained from the medical records. Serum albumin and CRP were measured using the modified bromocresol purple method and latex coagulating nephelometry, respectively. The NLR, LMR, PLR, CAR, PNI, and PINI were calculated as follows: total neutrophil count (/mm^3^) divided by the total lymphocyte count (/mm^3^), total lymphocyte count (/mm^3^) divided by the total monocyte count (/mm^3^), platelet count (/mm^3^) divided by the total lymphocyte count (/mm^3^), serum CRP (mg/dL) divided by serum albumin (g/dL), 10 × serum albumin (g/dL) + 0.005 × total lymphocyte count (/mm^3^), and 0.9 × serum albumin (g/dL) − 0.0007 × monocyte count (/mm^3^), respectively. These hematological markers were calculated based on blood tests conducted within 1 month from the first day of radiotherapy and within 1 week from the last day of radiotherapy. Pre- and post-treatment markers were termed as follows: pre-NLR, pre-LMR, pre-PLR, pre-CAR, pre-PNI, and pre-PINI for pretreatment markers and post-NLR, post-LMR, post-PLR, post-CAR, post-PNI, and post-PINI for post-treatment markers.

### Treatment

Radiotherapy was performed 5 days a week with a single daily fraction of 2 Gy using 4-MV X-ray linear accelerators applied to the primary tumor and cervical lymph nodes; intensity-modulated radiotherapy was utilized. The radiation dose for all the patients enrolled in this study was 70 Gy. Cisplatin (100 mg/m^2^/day, days 1, 22, and 43) was administered every 3 weeks during radiotherapy. All patients with nasopharyngeal, oropharyngeal, and hypopharyngeal cancer underwent percutaneous endoscopic gastrostomy after providing consent prior to treatment, unless there were anatomical problems in the stomach or abdomen. Patients were encouraged a dietary intake of at least 30 kcal/kg/day during treatment.

### Ethics

This study was approved by the ﻿Ethics Committee of Shinshu University School of Medicine ( approval number: 5619). ﻿Informed consent was obtained from all participants using an opt-out form. The study was performed in accordance with the principles outlined in the Declaration of Helsinki.

### Statistical analysis

The ROC curve for PFS was generated to establish the cutoff values of the pre- and post-treatment markers. OS was defined as the time from the date of diagnosis to the date of death from any cause. PFS was defined as the time from diagnosis to death or disease recurrence. The Kaplan–Meier survival curves of ﻿OS and PFS were compared using the log-rank test. Cox proportional hazards models were used to perform multivariate analysis using a backward stepwise selection method, to identify prognostic factors for OS and PFS. ﻿Differences were considered statistically significant at *p*-values < 0.05. All statistical tests were performed using the statistical software SPSS (version 28.0; SPSS Inc., Chicago, IL, USA).

## Supplementary Information

Supplementary Information.

## Data Availability

All data generated and analyzed in this study are available in this article.
