# Supplementary material for: Prognostic significance of pre- and post-treatment hematological biomarkers in patients with head and neck cancer treated with chemoradiotherapy
Source: Sci Rep. 2023 Mar 8;13:3869. doi: 10.1038/s41598-023-30584-1 (PMC9995483; doi:10.1038/s41598-023-30584-1)
Supplement: Supplementary file 1 — Supplementary Information. [file 41598_2023_30584_MOESM1_ESM.pdf]

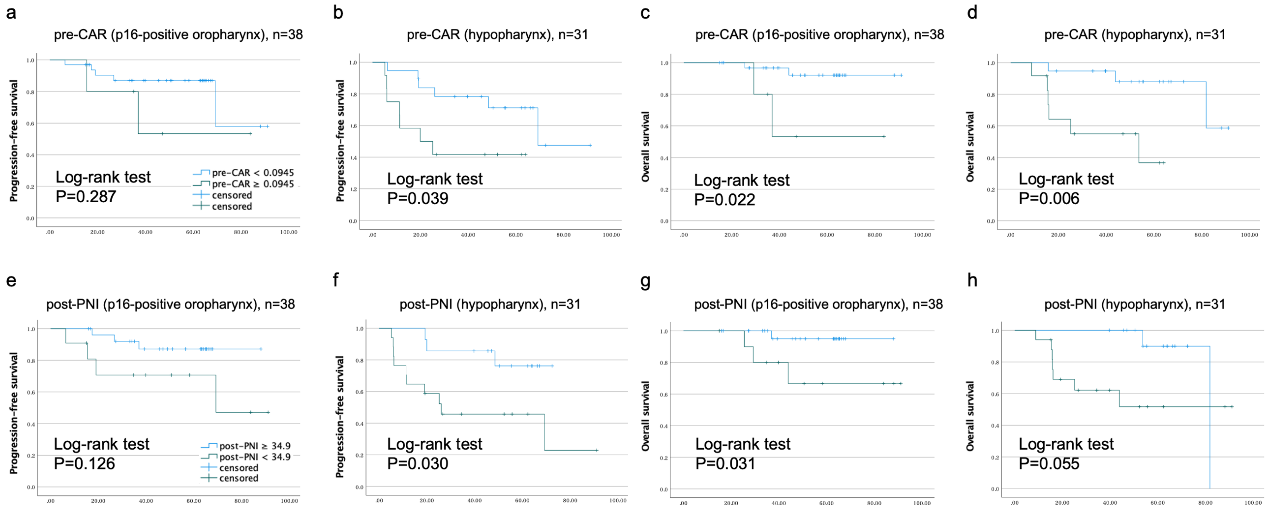

Figure S1. Kaplan–Meier curves for progression-free survival (PFS) and overall survival (OS) of p16-positive oropharyngeal and hypopharyngeal cancers according to the pre-CAR (a-d) and post-PNI (e-h)

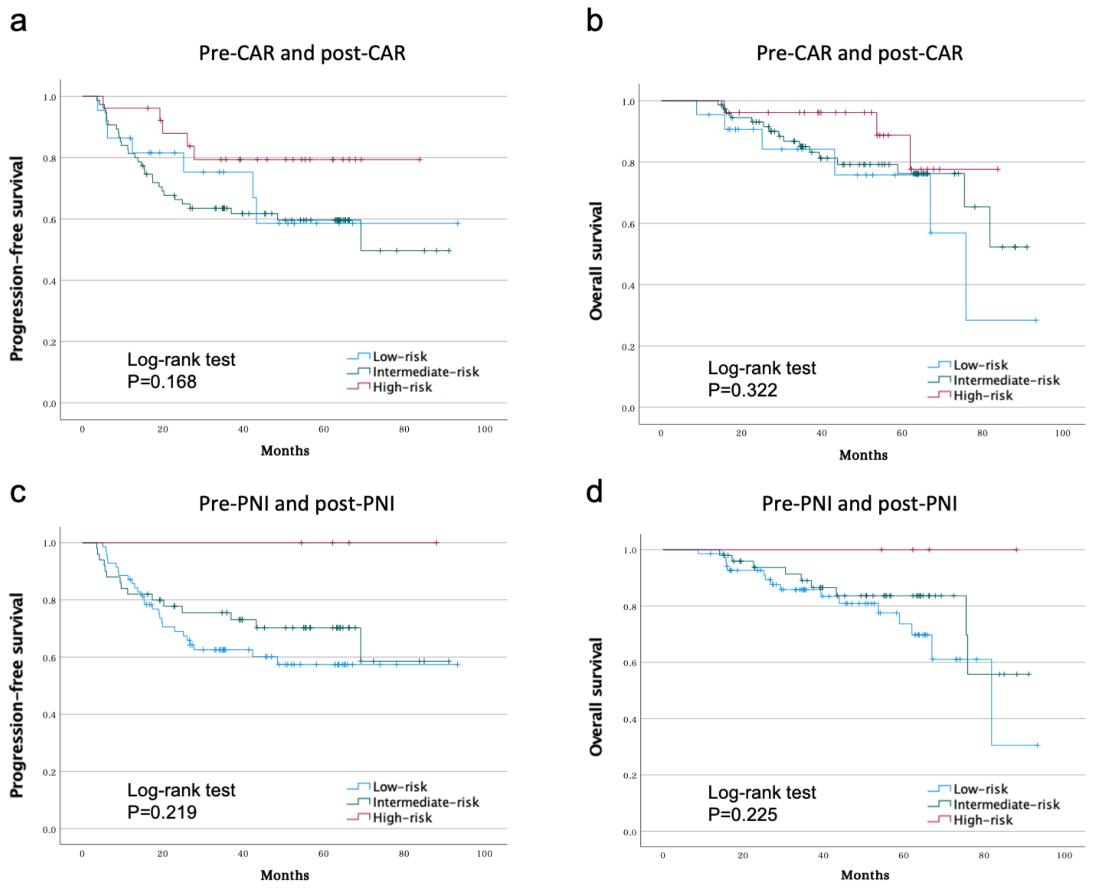

Figure S2. Prognostic investigation with respect to the changes in the CAR and PNI values before and after treatment

a, b) Kaplan–Meier curves for PFS and OS plotted according to the status of pre-CAR and post-CAR

Patients with both low pre-CAR and low post-CAR were assigned to the low-risk group, those with either high pre-CAR or high post-CAR were assigned to the intermediate-risk group, and those with both high pre-CAR and high post-CAR were assigned to the high-risk group.

c, d) Kaplan–Meier curves for PFS and OS plotted according to the status of the pre-PNI and post-PNI

Patients with both high pre-PNI and high post-PNI were assigned to the low-risk group, those with either low pre-PNI or low post-PNI were assigned to the intermediate-risk group, and those with both low pre-

PNI and low post-PNI were assigned to the high-risk group.
